# Care of the injured worldwide: trauma still the neglected disease of modern society

**DOI:** 10.1186/1757-7241-20-64

**Published:** 2012-09-15

**Authors:** Joseph V Sakran, Sarah E Greer, Evan Werlin, Maureen McCunn

**Affiliations:** 1 Department of Surgery, Medical University of South Carolina, 96 Jonathan Lucas Street (MSC 613/CSB 420), Charleston, SC, 29425-6130, USA; 2 Department of Surgery, Hospital of the University of Pennsylvania, 3400 Spruce Street, Maloney 5, Philadelphia, PA, 19104, USA; 3 Perelman School of Medicine, University of Pennsylvania, 3400 Spruce Street, Maloney 5, Philadelphia, PA, 19104, USA; 4 Department of Anesthesiology and Critical Care, Hospital of the University of Pennsylvania, 3400 Spruce Street, Dulles 6, Philadelphia, PA, 19104, USA

## Abstract

Traditionally, surgical diseases including emergency and injury care have garnered less attention and support internationally when compared to other medical specialties. Over the past decade however, healthcare professionals have increasingly advocated for the need to address the global burden of non-communicable diseases. Surgical disease, including traumatic injury, is among the top causes of death and disability worldwide and the subsequent economic burden is substantial, falling disproportionately on low- and middle-income countries (LMICs). The future of global health in these regions depends on a redirection of attention to diseases managed within surgical, anesthesia and emergency specialties. Increasing awareness of these disparities, as well as increasing focus in the realms of policy and advocacy, is crucial. While the barriers to providing quality trauma and emergency care worldwide are not insurmountable, we must work together across disciplines and across boundaries in order to negotiate change and reduce the global burden of surgical disease.

## Global burden of trauma and emergency surgical disease

Global support for surgical diseases including emergency and injury care has garnered less attention when compared to other medical specialties. Over the past decade however, healthcare professionals have increasingly advocated for the need to address the global burden of non-communicable diseases, which includes surgical care and its related specialties. The future of global health in low- and middle-income countries (LMICs) depends on a redirection of attention to diseases managed within surgical, anesthesia, emergency medicine and critical care specialties.

In 1966, the United States (U.S.) National Academy of Sciences published the influential report titled *Accidental Death and Disability: The Neglected Disease of Modern Society*, more commonly known as The White Paper. This landmark report was vital in the development of the emergency medical services system in the U. S. [1], leading to significant improvement in prehospital care, coordination of care within trauma systems, as well as a focus on functional rehabilitation. Trauma and emergency care systems have continued to mature over the past five decades in the U.S. and other highly-developed nations, in contrast to LMICs where such organization and infrastructure is virtually non-existent. This review specifically does not focus so much on short term interventions, and aims to discuss sustainable long-term interventions that will bolster the health infrastructure in LMICs.

### Disparities in access to surgical care

Surgical disease, including traumatic injury, is among the top causes of death and disability worldwide [2]. Though much of the literature on the global burden of surgical disease is based on modeling and subjective surveys, it is clear that significant disparities exist and that access to care in LMICs varies widely. In 2004, it was estimated that only 3.5% of 234 million major surgical procedures were performed on the world’s poorest 35% (those with health care expenditures < $100 per capita). In contrast, 74% of the surgeries were performed on the 30% of individuals from countries with health care expenditures > $1000 per capita [3].

Using modeling data from 192 World Health Organization (WHO) states, it was estimated that in countries with health care expenditures < $100 per capita, the rates of surgery were only 295 per 100,000 [3]. These estimations were confirmed in a retrospective study of 8 district hospitals across Tanzania, Uganda and Mozambique (countries with health care expenditures of 22, 44, and 21 dollars respectively), where the median annual rate of major surgical procedures was 25 per 10,000 [4].

### Disability due to surgical disease

The repercussions of poor access to surgical care are highlighted by the staggering impact of untreated surgical disease worldwide. Estimates suggest that 11% of the world’s disability adjusted life years, or DALYs (an estimation of years of healthy life lost due to disease or disability), are due to conditions that could be adequately treated with surgery [5]. The worldwide DALYs lost due to surgical disease are 27 per 1,000, and this burden is felt disproportionally by LMICs. The African continent reports 38 DALYs per 1,000 in comparison to the Americas which report 21 DALYs per 1,000, well below the worldwide average [5]. Due to variable reporting and registry quality, the true estimation of years of healthy life lost due to disease is likely even greater.

These global statistics are reflected locally in a study at a 90-bed hospital in Sierra Leone. Inpatient surgical care comprised only 1.7% of the hospital’s 8598 total contacts in a 3-month period. However, the appropriate delivery of surgical services averted the loss of 15% of the hospital’s DALYs during the same period. Basic outpatient surgical services such as suturing, fracture reduction or casting accounted for a significant portion of the hospital’s caseload [5].

### Injury and trauma

Traumatic injury is the leading cause of death under the age of 45 in the U.S. and worldwide [6]. Approximately 5.8 million people die each year as a result of injuries. This accounts for 10% of the world’s deaths, more than the number of fatalities from malaria, tuberculosis and HIV/AIDS combined. Furthermore, ninety percent of these injury deaths occur in LMICs [2]. Because trauma affects a relatively younger population, it accounts for more productive years of work lost than other illness, with an enormous economic and societal impact.

The global burden of injury is inversely proportional to income. Mortality increases concomitantly with the decreasing economic level of a country, from 35% mortality rates for injured patients (with Injury Severity Scores (ISS) > 9) in the U.S., to 55% in Mexico, to 63% in Ghana [7]. Eliminating these inequalities has the potential to result in 2,000,000 lives per year saved [7]. Injuries account for 63 million DALYs worldwide, with the highest per capita burden in Africa at 15 DALYs per 1,000 [5].

### Mechanism of injury

**Figure 1:**
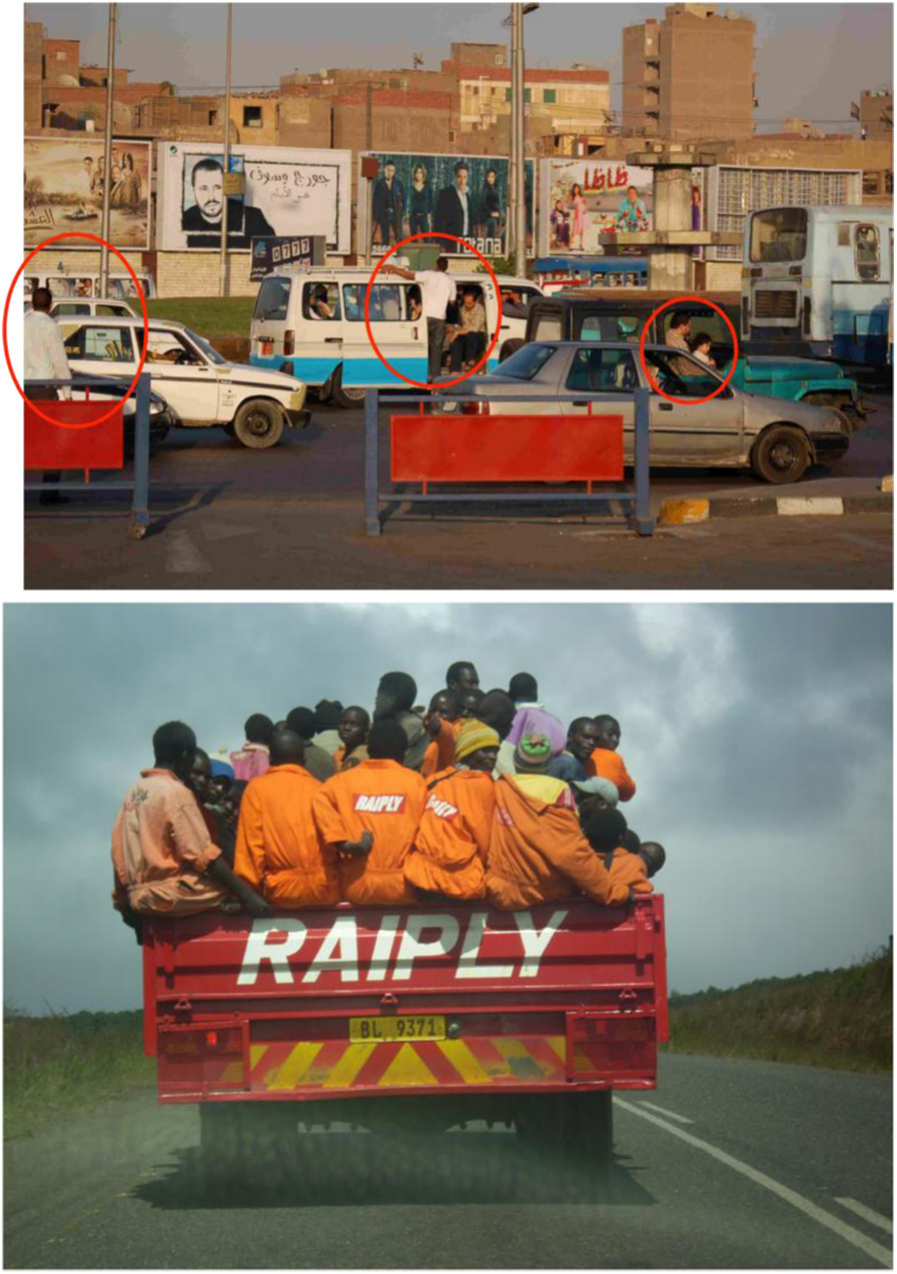
Traffic on two-lane road in Egypt (above), and Malawi (below) showing automobiles, pedestrians (lack of sidewalks or crosswalks), and absence of traffic signals or road signs.

The mechanism of injury leading to traumatic death (blunt, penetrating, drowning, burns) may vary by region, country and the relative state of war or peace locally. Road traffic crashes are the second highest cause of DALYs in 10–24 year olds, accounting for 5.4% of all DALYs; violence and self-inflicted injuries account for 3.5% and 2.8% respectively [8,9]. In countries with higher economic growth, access to motorized vehicles (automobiles, two- and three-wheelers) may exceed traffic system development: quality of roads, traffic signals, crosswalks, resulting in a higher rate of motor vehicle crashes. (See Figure 1) A number of LMICs have legislation in place for injury prevention (i.e. seatbelt or helmet law). However, the problem lies in enforcement of these laws: without the ability of many LMICs to enforce these laws, the prevention interventions become ineffective [10-13]. In 2004, traffic crashes accounted for 10% of all deaths globally in people aged 10–24 years [14]. With many of these crashes occurring in remote locations, a delay in care often occurs. Other barriers to getting patients immediate medical care are the actual terrain itself, (with most ground infrastructure being dirt roads riddled with pot-holes), lack of an organized pre-hospital transportation system, and inability to pay for transport to a hospital.

Violent injuries may be communal (war), inter-personal (assaults, firearms) or self-inflicted (poisoning, suicide). War and conflict leads not only to direct traumatic injury but also to injury more difficult to quantify, such as rape and torture [15], as well as the remnants of war, such as landmine injuries [16]. Patterns of injury seen in military conflicts [17-20] informs civilian care as new techniques for hemorrhage control and wound management are developed. Unfortunately these advances tend to occur primarily in higher-income countries where trauma system infrastructure already exists.

## Challenges to care

A number of factors in LMICs contribute to the significant morbidity and mortality associated with injury and emergency, and is reflective of the problems with access to care. Potential barriers to providing surgical care to the world’s poorest include a lack of effective political advocacy, insufficient resources (both personnel and consumables), and a perception that surgical intervention is not cost-effective by population-based measures.

### Prehospital care

In the prehospital setting, availability of transportation and lack of organized emergency medical services are the most significant impediments to providing optimal care. Just as overall mortality rates increase with decreasing economic status, prehospital death rates are also inversely proportional to economic status [7]. In a study comparing prehospital death rates, 59% of trauma deaths occurred in the field in high-income settings, compared to 72% in middle-income and 81% in low-income environments [7].

The time it takes to reach definitive care impacts this increased mortality rate. In most countries, there is no centralized “911” emergency call system and the ill or injured must transport themselves or pay for a private ambulance. Across 48 medium to large sized hospitals in Kenya, only 52% of patients arrived at the hospital within 30 minutes after a road traffic injury, and only 72% within one hour [21].

Several countries have adapted non-Western models of pre-hospital care [22] with training of “first responders” in the lay public [23-25] as well as the training of taxi drivers as *de facto* prehospital personnel [26-28]. Longitudinal studies that report outcomes after system implementation may help inform future infrastructure development. With a variety of systems and potential solutions, it is important to recognize that no single practice is applicable in all settings, but rather that prehospital care for emergency services should be adapted to meet local community needs [23,29-31].

### Hospital factors

The availability of international quality indicators is highly variable, and makes the objective assessment of care difficult. The WHO has played a prominent role in addressing this gap, and recognizes three categories pertaining to the provision of care where improvement efforts may be focused: physical resources (lack of equipment, or equipment that is not appropriately maintained), organizational resources (lack of performance improvement efforts), and human resources (lack of adequately trained staff). In 2004, the WHO working group publication *Guidelines for Essential Trauma Care* delineated 11 core essential services as well as 260 individual items (both physical and human resources) that should be available based on the level of care from small rural clinics to tertiary centers [32]. This publication, with its endorsement by the International Association of Trauma Surgery and Intensive Care (IATSIC), the International Trauma Anesthesia and Critical Care Society (ITACCS) and the International Society of Surgery, serves not only as a guideline for healthcare providers, but as an advocacy statement to prompt action [33,34].

### Physical resources

The harsh reality of economics impacts in-hospital trauma and surgical care in a very direct fashion. Cash deposits are frequently demanded prior to treatment, and often require the signing of a binding agreement [21]. This practice is not uncommon in many LMICs where injured patients must provide proof of payment prior to receiving even emergency care. Patient and families are often required to procure their own medical supplies, including not only medications, but also bandages and surgical dressings [31].

Physical resources must be not only available but functional. Life-saving and disability-preventing surgical procedures can be achieved only in conjunction with appropriate anesthesia services. In a convenience sampling of 22 countries and a total of 590 facilities, 35% of hospitals had no access to oxygen and 40% had no anesthesia machines Approximately 25% of facilities reported that they had no access to emergency airway equipment (laryngoscopes, endotracheal tubes, face masks) [35].

### Organizational resources

Trauma surgery has a long history of incorporating performance improvement efforts into clinical practice, but many LMIC do not have this organizational infrastructure in place. The WHO provides guidelines for trauma care resources through the *Global Initiative for Emergency and Essential Surgical Care*[36] and *Guidelines for Essential Trauma Care*[32] which have been implemented in several countries. Implementation of basic performance improvement efforts to identify preventable deaths and address patient care issues has been shown to decrease mortality, such as when implemented at hospital in Khon Kaen, Thailand [37]. The importance of establishing and attaining benchmarks is a better understanding of the processes of patient care, and lies at the foundation of improving quality of care for all patients. Another effort to bridge the knowledge gap is on-line resources that provide free educational material tailored towards LMIC such as: http://www.primarytraumacare.org, http://www.trauma.org.

### Human resources

There is a critical shortage of healthcare workers in LMICs. Disparities between global healthcare needs and the available workforce are due to multiple factors: lack of training, inadequate salary reimbursement, perceptions of a lack of professional status, and the ‘brain drain’ to more highly developed countries. Sub-Saharan Africa carries 24% of the global burden of disease but as little as 3% of the world’s health workers ( http://www.who.int/surgery/globalinitiative/en/). Many trained and educated nationals who work in the healthcare field leave their home country for better pay, more opportunities or better education for their families, and in conflict zones, often to escape the risk of imprisonment or death for treating enemy forces.

This leaves local hospitals often unable to perform procedures at the desired level. In district hospitals within the war-ravaged zones of Sri Lanka, 35.7% of the hospitals were unable to perform removal of a foreign body, 95% were unable to perform cricothyroidotomy or tracheostomy, and 60% were unable to perform chest tube insertion or burn management, opting instead to refer patients to other hospitals [38].

An issue that has been highlighted more recently is the fact that surgeons training in western societies continue to narrow their expertise as they become sub-specialized. One might see how this can spiral into a tremendous issue for LMICs that already deal with insufficient general surgery experts. This further supports training of non-physician providers that will be discussed in more detail below. In addition to the need for surgical expertise, the need for better anesthesia care and more anesthesiologists in LMICs is increasingly recognized as a healthcare concern. The World Health Assembly established a Task Force for Scaling Up Education and Training for Health Workers and identified several factors such as those listed above necessary for successful scale-up programs [39].

The high mortality rate associated with anesthesia in LMICs (as high as 1 death per 144 cases) is often ascribed to lack of training and oversight [40]. A recent survey to assess the availability of anesthesia providers in LMICs obtained information from Zambia, Yemen, Tanzania, Zimbabwe, Afghanistan, Senegal, Cameroon, Benin, the Democratic Republic of Congo, Rwanda, Uganda, and Cote d’Ivoir; Swaziland was the only country with more than one physician or non-physician anesthesia provider per 100,000 people [41-43]. A WHO survey tool administered to 344 health care facilities in LMICs showed 30% had no anesthesia provider in the medical facility, and 41% of anesthetics were delivered by uncertified, unlicensed and unregistered non-physician providers [44].

### Interventions

Of the 234 million surgical procedures performed worldwide, humanitarian aid organizations provide 50–100,000 surgical interventions annually [45]. While the presence of foreign nationals may help to support a healthcare system for the short term, in the long term, capacity building and efforts to create sustainable programs to address unmet trauma, emergency, and anesthesia healthcare needs is mandatory. Several highly-developed nations have paired with LMICs to create training programs, establish universities, sustain hospital services, run trauma and emergency care courses, and organize exchange programs that allow practitioners to work abroad [46-49].

### Non-physician providers – ‘task shifting’

Several countries have utilized the concept of task shifting in order to use healthcare workers replace or supplement the work-shortage of physicians, particularly in rural areas. A recent review identified 31 studies from various countries that have instituted task shifting through the use of non-physician providers, suggesting this is a promising policy option to increase productivity in provision of health care services as well as standardize the services provided at a given quality and cost [50]. This has been successfully implemented for trauma surgery in Cambodia [51]. A focused discussion of surgery and anesthesia needs in Africa also identified the value of task shifting, where surgical procedures and anesthetics are performed by other providers when physicians are not available [52].

### Additional training

The American College of Surgeons Advanced Trauma Life Support (ATLS®) course offers a basic foundation in the principles and practice of trauma care, but with significant impact. Institution of ATLS in Trinidad was associated with a decrease in mortality from 67% to 34% for severely injured patients [53,54]. Other efforts to provide additional training as well as further opportunity for exchange between surgeons across countries have demonstrated benefit [55,56]. Commonalities in knowledge, training, and previous experiences will allow for formation of interdependent multidisciplinary teams. Trauma team training, as occurs in the military and in high-income nations, can also be implemented in hospitals in LMICs [57].

Global anesthesia outreach in education and local training of anesthesia providers is a short-term strategy supported by the WHO to improve the quality and quantity of providers in countries with insufficiently met needs [41]. The sustained presence of Western-trained anesthesiologists, in rotation or for several months’ time, could contribute to the training and education of providers in developing nations. Support for LMICs to develop their own education and training, to institute programs for the specialty of anesthesia, and to adopt the practice of ‘task shifting’, would help to lessen the gap between need and availability of qualified providers. An approach to addressing global health care needs is seen in the Medical School for International Health, a partnership between Ben-Gurion and Columbia universities. This unique program prepares students to work both in medicine and an international health setting, emphasizing the practice of preventive and population based medicine across cultures [58].

## Future directions

The global burden of trauma and emergency surgical disease is vast and includes anesthesia services; pre-hospital systems; and physical, human and organizational resource availability. There is a growing recognition among health care providers however, that the future of global health in low- or middle-income countries (LMICs) depends not only on addressing communicable diseases, but focusing attention on diseases managed within surgical, anesthesia, and emergency care specialties. Increasing the awareness of existing disparities, as well as stakeholder involvement in the realms of policy and advocacy is vital to improving the current situation. The barriers to providing quality trauma and emergency care worldwide are not insurmountable – but we must work together across disciplines and across borders if we are to raise the bar and negotiate change. Our patients deserve no less.

## Competing interests

The authors declare that they have no competing interests.

## Authors’ contributions

Design – JS, SG, EW, MM Literature Review – JS, SG, EW, MM Analysis of Data – JS, SG, EW, MM Writing of Manuscript – JS, SG, EW, MM All authors read and approved the final manuscript.
